# A Single-Step Method for Rapid Extraction of Total Lipids from Green Microalgae

**DOI:** 10.1371/journal.pone.0089643

**Published:** 2014-02-24

**Authors:** Martin Axelsson, Francesco Gentili

**Affiliations:** Department of Wildlife, Fish, and Environmental Studies, Swedish University of Agricultural Sciences, Umeå, Sweden; RIKEN PSC, Japan

## Abstract

Microalgae produce a wide range of lipid compounds of potential commercial interest. Total lipid extraction performed by conventional extraction methods, relying on the chloroform-methanol solvent system are too laborious and time consuming for screening large numbers of samples. In this study, three previous extraction methods devised by Folch et al. (1957), Bligh and Dyer (1959) and Selstam and Öquist (1985) were compared and a faster single-step procedure was developed for extraction of total lipids from green microalgae. In the single-step procedure, 8 ml of a 2∶1 chloroform-methanol (v/v) mixture was added to fresh or frozen microalgal paste or pulverized dry algal biomass contained in a glass centrifuge tube. The biomass was manually suspended by vigorously shaking the tube for a few seconds and 2 ml of a 0.73% NaCl water solution was added. Phase separation was facilitated by 2 min of centrifugation at 350 g and the lower phase was recovered for analysis. An uncharacterized microalgal polyculture and the green microalgae *Scenedesmus dimorphus, Selenastrum minutum*, and *Chlorella protothecoides* were subjected to the different extraction methods and various techniques of biomass homogenization. The less labour intensive single-step procedure presented here allowed simultaneous recovery of total lipid extracts from multiple samples of green microalgae with quantitative yields and fatty acid profiles comparable to those of the previous methods. While the single-step procedure is highly correlated in lipid extractability (r^2^ = 0.985) to the previous method of Folch et al. (1957), it allowed at least five times higher sample throughput.

## Introduction

Oleaginous microalgae have received considerable attention as a renewable source of oil for production of biodiesel and other fuels [1]. Some microalgal lipids are already valued ingredients in aquaculture feeds [2], [3] and as nutritional supplements for humans [4], [5]. However, microalgae are known to synthesize a diversity of unusual lipid compounds which may be commercially exploitable [6], [7], [8] and have been proposed as a suitable biorefinery feedstock for value added co-production of fine chemicals and fuels [9]. While specific analytical methods may exist for selected lipid compounds, total lipid extraction is favourable when screening for a variety of lipids. The extraction methods devised by Folch et al. [10] and by Bligh and Dyer [11] have found general acceptance as standard procedures for recovery of total lipids [12], [13]. Both methods rely on chloroform and methanol to form a monophasic solvent system to extract and dissolve the lipids. A biphasic system is then produced in a purification step by the addition of water, leading to the separation of polar and non-polar compounds into an upper and lower phase respectively [10], [11].

The method by Folch et al. [10] is regarded as the most reliable method for complete recovery of total lipids but the Bligh and Dyer [11] procedure is more widely known [14] and has been favoured for extraction of lipids from tissues of vascular plants [15]. It is also the more commonly used method but has often been incorrectly applied resulting in incomplete recovery [12]. While fatty acid profiles may remain intact, the method systematically underestimates concentrations in samples containing more than 2% lipids [14]. This limitation is important to consider in microalgal research as oleaginous microalgae contain an average of 25.5% lipid by dry weight during normal circumstances and 45.7% when subjected to stress [16].

The main differences between the protocols of Folch et al. [10] and Bligh and Dyer [11] are the volume of solvent system in relation to the amount of sample, the ratios between solvents within the systems, and the presence or absence of NaCl in the added water fraction. While Folch et al. [10] employed 20 times the sample volume of a 2∶1 (v/v) chloroform-methanol mixture (assuming that the tissue had the specific gravity of water), Bligh and Dyer [11] used a chloroform-methanol step wise extraction of 1∶2 and 1∶1 (v/v) amounting to a final volume of only four times the equivalent sample amount. Other solvent systems have been developed as alternatives to the toxic chloroform systems, but these are generally less efficient for total lipid extraction and are sensitive to the water content of the sample [15], [17]–[20]. The conventional chloroform based methods are considered superior for total lipid extraction, but they are also notorious for being laborious and time consuming, thus limiting sample throughput. The protocols generally involve sequential addition of solvents and several steps of manual sample manipulation, such as homogenization and filtration, making them unsuitable for screening large numbers of samples.

The aim of this study was to a) compare three established total lipid extraction methods relying on the chloroform-methanol solvent system and b) to find a faster and simpler procedure to simultaneously obtain extracts from multiple small samples of green freshwater microalgae (a few hundred mg of wet weight biomass). Presented here is the development of a one-step total lipid extraction procedure to facilitate screening for quantitative total lipid yields and fatty acid compositions among green freshwater microalgae.

## Methods

### Ethics statement

“N/A”.

No specific permissions were required for these locations/activities because we used local species or species present naturally in our environment. We confirm that the field studies did not involve endangered or protected species.

We had the permission to collect our samples from the private land at the power plant.

### 1. Cultivation and harvest of microalgae

The green microalgal strains *Scenedesmus dimorphus* (417), *Selenastrum minutum* (326) and *Chlorella protothecoides* (25) were purchased from UTEX The Culture Collection of Algae at the University of Texas at Austin (in parenthesis is the UTEX id); while an uncharacterized polyculture of algae cultured in municipal wastewater, was retrieved from a bioreactor installed at a combined heat and power plant (Umeå Energi, Umeå).

For the lipid extraction method comparison, two independent experiments were carried out. In experiment 1, *S. dimorphus* (417), S. *minutum* (326) and *C. protothecoides* (25) were grown in Proteose medium [21] and the polyculture of endogenous algae was cultured in untreated final municipal wastewater treatment plant effluent. All cultures were grown in bottles with 1 liter of volume sparged with sterile air at a flow of 170 ml/min and subjected to approximately 12 hours of natural light from a window in Umeå, Sweden (March to April, 2012, 63°49′30′′N). After four weeks of growth, biomass was harvested by centrifugation at 3584 g for 10 min and pellets were stored at −20°C overnight. Experiment 1 had two replicates.

In experiment 2, the same algae were grown in untreated municipal wastewater treatment plant influent. All cultures were grown in bottles of 1 liter of volume sparged with flue gases at a flow of 170 ml/min containing approximately 10% CO_2_, from a combined heat and power plant (Umeå Energi, Umeå). *S. dimorphus* (417), *S. minutum* (326) and the algal polyculture were grown for four days while *C. protothecoides* was grown for 11 days in a greenhouse in Umeå in April 2013 at an average temperature of 19°C, receiving approximately 16 hours of natural light a day at an average PAR (photosynthetic active radiation) intensity of 715 µEm^−2^s^−1^. The PAR was measured and recorded every 5 minutes using a LiCor 1400 datalogger connected to a spherical light sensor LI 193 (LiCor Lincoln, Nebraska USA). The algae were harvested as mentioned in experiment 1 and immediately subjected to lipid extraction. Experiment 2 had four replicates.

For the cell disruption method comparison, the same algae were cultured in autoclaved (121°C, 20 min.) municipal wastewater treatment plant effluent. The growth conditions were the same as described for experiment 1.

For the oven dried experiment, biomass was harvested from an uncharacterized polyculture of microalgae grown in a 650 l bioreactor with municipal wastewater and treated flue gas, 3 l/min containing approximately 10% CO_2_, from a combined heat and power plant (Umeå Energi, Umeå). The bioreactor was placed in a greenhouse on the roof of the combined heat and power plant. The algal polyculture was grown under a batch regime in February 2012 at an average temperature of 19°C receiving approximately 10 hours of natural light a day at an average PAR (photosynthetic active radiation) intensity of 361 µEm^−2^s^−1^. Samples of algae, 12.73±0.31 mg (mean ± SE) dry weight harvested in the morning, were pelletized by centrifugation at 3584 g and stored overnight at −20°C.

### 2. Lipid extraction: Previous methods

All reagents were of analytical grade, chloroform was purchased from VWR and methanol from Fischer-Scientific.

The protocols of Folch et al. [10], Bligh and Dyer [11], and Selstam and Öquist [22], the latter based on the method of Bligh and Dyer for vascular plant material, were adapted to provide a final solvent system volume of 10 ml for extraction of about 200 mg of wet weight microalgal biomass. All weight measurements were done with a high precision balance (Kern ABT 120-5DM; readout 0.01 mg; Kern, Germany). Microalgal paste was homogenized in a glass Potter-Elvehjelm homogenizer together with solvents. Cell debris was removed by means of vacuum filtration through a Whatman grade GF/C glass microfiber filter (1.2 µm) into a glass centrifuge tube. Phase separation was facilitated by centrifugation at 350 g for 2 min and the organic, lower phase was placed in an aluminium foil cup for overnight solvent evaporation at room temperature followed by gravimetrical determination of the lipid extract.

In the Folch method, microalgal paste was homogenized in a 2∶1 chloroform:methanol (v/v) mixture and cell debris was removed by filtration. The homogenizer and collected cell debris were rinsed with fresh solvent mixture and the rinse was pooled with the previous filtrate prior to the addition of a 0.73% NaCl water solution, producing a final solvent system of 2∶1∶0.8 chloroform:methanol:water (v/v/v).

In the method of Bligh and Dyer, microalgal paste was mixed with deionized water, chloroform, and methanol to reach 1∶2∶0.8 parts chloroform:methanol:water (v/v/v) and homogenized. One part chloroform was added and the mixture was further homogenized. Then, one part deionized water was added to the homogenate giving a final ratio of 2∶2∶1.8 chloroform:methanol:water (v/v/v); the homogenate was re-homogenized and finally filtered to remove cell debris.

In the Selstam and Öquist procedure, microalgal paste was homogenized in chloroform and a 4∶1 mixture of methanol and 0.73% NaCl water solution producing a 1∶2∶0.5 chloroform:methanol:water (v/v/v) system. The homogenate was filtered and the homogenizer and cell debris rinsed with fresh methanol-water mixture and chloroform resulting in 2∶3.6∶0.9 parts chloroform:methanol:water (v/v/v) as the rinse was collected to the previous filtrate. Finally, more chloroform and 0.73% NaCl water solution were added to give a ratio of 1∶1∶0.8 chloroform:methanol:water (v/v/v).

### 3. Lipid extraction: Single-step procedure

Microalgal paste was resuspended in 2∶1 parts of chloroform:methanol (v/v) by manually shaking the tube vigorously for a few seconds or until the biomass was dispersed in the solvent system. Finally a 0.73% NaCl water solution was added to produce a 2∶1∶0.8 system of chloroform:methanol:water (v/v/v).

### 4. Extraction of lipids from oven dried microalgae

Two quadruplicate sets of frozen pelletized algae (for cultivation details see 2.1 Cultivation and harvest of microalgae) were placed in tin foil cups and dried at 65°C overnight. Lipid extractions were performed from the dried biomass without cell disruption or using a glass Potter-Elvehjelm homogenizer and from frozen pellets according to the single-step procedure.

### 5. Cell disruption

Different methods of cell disruption, allowing simultaneous treatment of several samples, were assessed as alternatives to the Potter-Elvehjelm homogenizer in the single-step extraction procedure. Grinding in liquid nitrogen and ultrasonication using a probe were not investigated as they would not allow simultaneous treatment of multiple samples. The microalgal paste was subjected to either 1) no treatment, 2) Potter-Elvehjelm homogenization, 3) microwaves at full effect (557 W) for 1 min followed by low effect (254 W) for 4 min, 4) microwaves at full effect (557 W) for 3 min or 5) ultrasonication for 30 min in a sonicator bath (47 kHz, 60 W, Branson B-2200). The microwave treatments were performed prior to the addition of solvents, the Potter-Elvehjelm homogenization was performed in 4 ml of the solvent mixture following a rinse with the same amount while the samples of the other treatments were directly subjected to 8 ml of the 2∶1 chloroform-methanol (v/v) solvent mixture. Post treatment, extracts were washed with 2 ml 0.73% NaCl water solution and recovered as previously described.

### 6. Chemical analysis of lipid extracts

For a general screening of extracted compounds, solvents were removed by evaporation and the dry extracts were dissolved in dichloromethane prior to a full scan analysis by GC/MS for m/z fragments up to 700 (DB50 column, EI 35 eV, quadrupole).

Qualitative and quantitative FA (fatty acids) profile analyses were performed at the Department of Food Science, Swedish University of Agricultural Sciences (SLU) Uppsala, Sweden. FA were methylated as previously described [23] and analyzed with a gas chromatograph with a FID detector (GC-FID) [24]. The GC-FID analyses were done with two and three replicates for qualitative analyses (experiment 1 and 2) and with three replicates with internal standards (461 standard reference mixture from Nu-Chek Prep Ink. USA) for quantitative analyses (experiment 2).

### 7. Statistical analysis

Data were analyzed at a 95% confidence level either using a two-sample t-test or one-way analysis of variance (ANOVA) with Tukey's post-hoc test and by regression analysis (Minitab 16.1.0).

## Results and discussion

The methods of Folch et al. [10], Bligh and Dyer [11], and Selstam and Öquist [22] were compared, adapted and evaluated prior to selecting a method suitable for a rapid and simple procedure for simultaneous extraction of multiple samples.

### 1. Comparison of the previous methods

For optimum lipid recovery, the order at which the individual solvents are added is important [25]. Yields were indeed significantly smaller if water was added prior to the methanol and chloroform mixture in the single-step procedure (data not shown). In addition, the endogenous water of the sample must be taken into consideration while performing an extraction as it should mix with the chloroform and methanol to form a monophasic ternary system [11]. The water content of wet microalgal paste is notably high and was accounted for in this work. The increased solvent-to-sample ratios assumed here would however reduce the importance of this factor as the capacity of the solvent system for retaining the monophasic system increases with its volume [14]. Increased solvent-to-sample ratios should make the extraction system more robust and allow more variation in sample content and size. In the present study, twice the volume of final solvent system used by Folch et al. [10] and almost nine times the volume used by Bligh and Dyer [11] were employed. Bligh and Dyer wanted to avoid large solvent volumes but in the present work their solvent-to-sample ratio would result in an inconveniently small volume. A larger solvent-to-sample ratio is also justified in view of the reported limitations of the Bligh and Dyer procedure when employed on lipid rich samples [14], [26].

**Figure 1:**
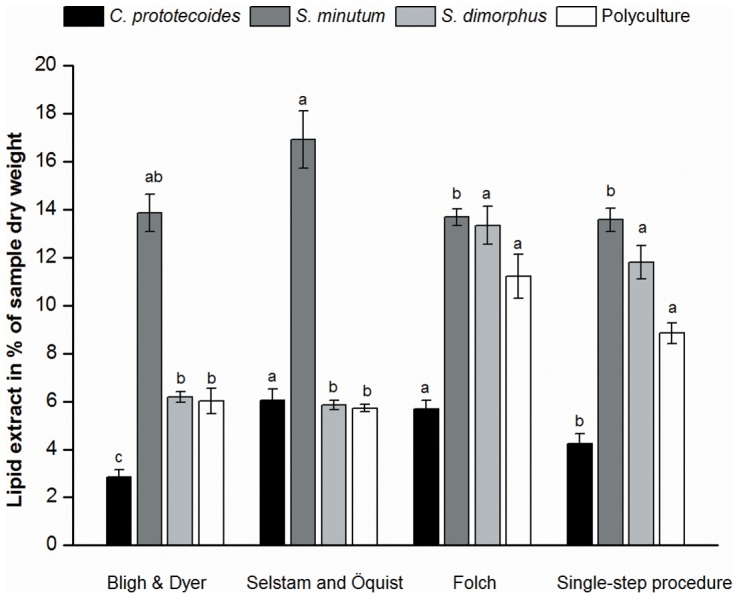
Gravimetric yields of total lipids extracted by the previous methods and the single-step procedure. Bars show mean yields from the different algae expressed as a percentage of the algal sample dry weight (mean ± SE, n = 4; experiment 2). Different letters above bars of the same alga indicate a significant difference at α = 0.05.

The method of Folch et al. [10] was easier and faster to perform as it involved less sample manipulation compared to the other previous methods. In addition, with the exception of *S. minutum*, the Folch method resulted in significantly higher gravimetrical yields of extracted lipids compared to the other two methods (Fig. 1).Thus, the method of Folch et al. [10] was selected as a basis for further development. While performing extractions using the previous methods, homogenization and subsequent filtration of the solvent-sample system were recognized as particular impediments to simultaneous extraction of many samples.

### 2. Filtration and multiple step extractions

A filtration step to remove obstructive tissue from the sample was employed in the method by Folch et al. [10] to allow efficient phase separation and recovery of the lipid fraction. However, in the present study, the amount of sample in relation to solvent volume was not large enough to be obstructive (230±35 mg wet weight biomass was extracted in 10 ml solvent system). Debris from the biomass sample formed a thin, distinct layer between the two phases and the lower phase was easily accessible using a glass Pasteur pipette. If handled gently the small layer of debris did not readily mix with the chloroform and the lower phase could easily be recovered without prior filtration.

Multiple step extraction, i.e. to repeatedly rinse the sample with fresh solvent, is commonly performed to increase yields but single-step extraction has been shown to achieve equally high or only slightly smaller recoveries [26], [27]. To save time and conserve resources, only single-step extractions were carried out in the present study.

### 3. Comparison of techniques for cell disruption

Manual homogenization of individual samples was the most time consuming step of the extraction procedure. While Bligh and Dyer [11] employed a blender, Folch et al. [10] used a Potter-Elvehjelm homogenizer. The latter is widely used for small scale homogenization of suspended cell cultures, but any means of homogenization should suffice. The aim of Folch et al. and Bligh and Dyer was to extract lipids from more or less solid animal tissues [10], [11]. Plant tissues are generally more rigid and may require homogenization down to a particle size of 300 µm or smaller [13]. Unicellular microalgae are already small in particle size but neutral lipids, most notably triacylglycerols stored within the algae, could nonetheless be solubilized by disrupting the cells. However, while employing a chloroform-methanol solvent system, complete cell disruption is not needed as the lipids are extracted across the cell wall [28]. Nevertheless, a cell disruptive treatment step could still have an impact on lipid extractability and yield from microalgae [28].

**Figure 2:**
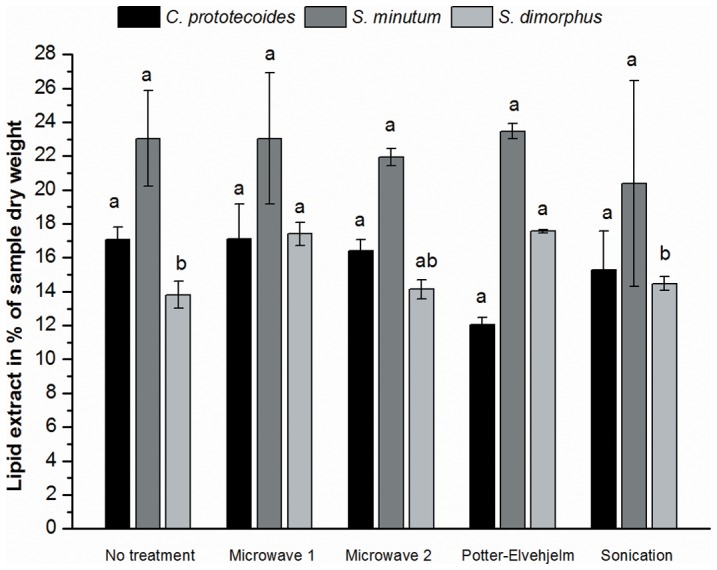
Comparison of cell disruption techniques to increase yields. Bars show gravimetric yields of total lipids extracted by the single-step procedure from different algal species. Yields are expressed as a percentage of the algal sample in dry weight (mean ± SE, n = 2; experiment 1). Different letters above bars of the same alga indicate a significant difference at α = 0.05.

In this study, different methods of cell disruption allowing simultaneous treatment of samples were assessed as alternatives to the Potter-Elvehjelm homogenizer. Microwave treatments have previously been employed to facilitate solvent extraction of lipids [29], [30] and as a means to permeabilize the thick and rigid cell walls of green microalgae for staining purposes [31]. Lee et al. [30] investigated different methods of cell disruption for solvent extraction of lipids from green microalgae and acquired the highest yields when microwave treatment was employed. Though microwave cell disruption techniques are relatively novel, ultrasonication is a proven and popular method for disruption of microalgal cells [32] and may be the preferred method of disruption for protein extraction [33]. However, none of the investigated cell disruption techniques produced substantially higher yields (Fig. 2) and the only differences of statistical significance were achieved when treating *S. dimorphus* with microwaves or the Potter-Elvehjelm homogenizer, which increased yields by approximately 24% compared to the untreated control and to sonication (Fig. 2). In addition, Ryckebosch et al. [27] reported that employing a similar total lipid extraction method could slightly increase yields from *S. obliquus* by cell disruption, but concluded that no cell disruption was generally necessary for lipid extraction from lyophilized microalgae. Indeed, none of the cell disruption methods used in this study provided a general gain in yields.

### 4. The single-step procedure

Yields of the single-step procedure closely resembled those of the previous Folch method (Fig. 1 and in experiment 1 from frozen algae biomass data not shown). To investigate if the modification to the previous protocol affected the relative recovery of individual lipid compounds, fatty acid profiles of extracts from *S. minutum* were determined and a semi-qualitative GC/MS analysis was employed in full scan mode to screen for undefined compounds. Between the single-step procedure and the previous method, there were no differences in presence of undefined compounds determined by overlay analysis of chromatograms normalized against sample concentrations and matched according to retention time. Quantitative FA profiles, obtained from *S. minutum* using the single-step procedure, were compared by regression analysis to those obtained using the previous method by Folch et al. [10]. The profiles of the single-step procedure closely resembled those of the previous method, producing a regression coefficient (r^2^) of 0.985. Qualitative FA profiles had a regression coefficient r^2^ equal to 0.991 when comparing the most representative fatty acids extracted (Table 1 and experiment 1 data not shown). Also qualitative FA profiles between the single-step procedure and the methods by Bligh and Dyer [11] and Selstam and Öquist [22] were highly correlated (Table 1) with a regression coefficient r^2^ equal to 0.99 and 0.992 respectively. Furthermore, the single-step procedure produced the same gravimetrical yields of total lipids from dried microalgae as from fresh or frozen microalgal paste if the dried biomass was homogenized before or during the extraction.

**Table 1:** The fatty acid profile of *Selenastrum minutum* in extracts obtained by the different methods.

	Bligh and Dyer			Selstam and Öquist			Folch et al.			Single-step		
		%			%			%			%	
C16:0	23.86	±	0.43	24.26	±	0.66	25.05	±	0.43	23.92	±	0.19
C18:0	1.46	±	0.18	1.49	±	0.08	1.77	±	0.13	1.26	±	0.06
C18:1	20.43	±	0.24	21.49	±	0.20	20.68	±	0.17	19.62	±	0.15
C18:2	9.47	±	0.11	9.04	±	0.22	7.64	±	0.12	7.77	±	0.06
C18:3	38.46	±	0.56	37.53	±	0.70	37.17	±	0.39	40.06	±	0.25
C24:0	2.35	±	0.09	2.23	±	0.13	3.47	±	0.26	3.45	±	0.05

Presence of the most representative fatty acids in total lipid extracts of *Selenastrum minutum* obtained by the previous extraction methods and by the single-step procedure developed in this study. Individual FA presented in per cent of total FA (mean ± SE, n = 3; experiment 2).

To validate the recovery of total lipids using the single-step procedure, a known amount of vegetable oil (olive oil; 1.2 – 1.8 mg) was added to the extracting solution.

As determined gravimetrically, the procedure achieved complete recovery of the vegetable oil, showing an average recovery of 91%±4.7 SE.

In an additional experiment performed on quadruplicate samples the single-step procedure had 3.5 times higher total lipids yield than a method based on hexane extraction [19] for the green alga *S. minutum* (data not shown).

In a recent study an extraction methodology similar to the single-step procedure but favouring a system of 1∶1 (v/v) chloroform-methanol was proposed for extraction of lipids from microalgae [27]. This ratio was investigated and compared to the 2∶1 (v/v) ratio with the same setup as in the single-step procedure. Gravimetrical yields appeared significantly higher but blank control extractions showed that the 1∶1 system left residues after evaporating the solvents. The remnants could not be explained by any isolated individual constituent of the solvent system. Note that the 2∶1 system evaluated in this paper, employing the same constituents, did not leave significant residues.

The gravimetric yield of the single-step procedure was however dependent on the sample size relative to the solvent system volume as extraction from larger sizes of sample resulted in smaller relative yields. A sample size limit assessment of the 10 ml solvent system revealed that up to 300 mg of wet microalgal paste corresponding to ca 30 mg in dry weight could be extracted (data not shown). This limitation was less pronounced when extracting lipids from dried biomass which allowed a 3 times larger maximum sample size (i.e. 90 mg). Below these limits, sample size has a small relative effect on gravimetric yield but lipid profiles should remain intact. Nevertheless, for mutual comparisons, samples should be normalized with regard to size prior to extraction to avoid inaccurate results.

Only green freshwater microalgae were assessed in this study and the presented method should be used with caution in works on other microalgae.

## Conclusions

The single-step procedure is suitable for total lipid extraction and may be applied fo screening of algae for qualitative-quantitative analyses of total fatty acids. The method presented in this work had at least five times higher sample throughput when compared to the previous methods.
